# Exploring the Enablers and Barriers to Healthy Eating for Lactating Mothers in Underserved Settings in Ghana

**DOI:** 10.1016/j.cdnut.2026.107636

**Published:** 2026-01-13

**Authors:** Maximillian Kolbe Domapielle, Mildred Naamwintome Molle, Rudolf Abugnaba-Abanga

**Affiliations:** 1 Department of Governance and Development Management, Faculty of Public Policy, and Governance, University of Business Integrated Development Studies, Wa Bamahu, Ghana; 2 Department of Geography and Environment, University of Western Ontario, London, ON, Canada; 3 Department of Climate Change and Health, West African Center for Sustainable Rural Transformation (WAC-SRT), Climate Change and Health Division, University of Business and Integrated Development Studies, Wa Bamahu, Ghana

**Keywords:** breastfeeding, barriers, enablers, healthy eating, lactating mothers, nutrition

## Abstract

**Background:**

Optimal nutrition for lactating mothers has remained at the top of the health policy agendas of many developing countries because of the consensus that it is an effective channel for achieving positive maternal and child health outcomes.

**Objectives:**

This study draws on the socio-ecological theory to explore barriers and enablers of healthy eating among lactating mothers in the Wa West District of Ghana. Previous studies tended to focus on the barriers and merely experimented with the enablers and opportunities for engraining and sustaining healthy eating for lactating mothers in underserved settings.

**Methods:**

We employed an exploratory case study design and used convenience sampling procedures to select 30 lactating women and purposive sampling to select 6 midwives and 2 nutrition officers to participate in the study. Semi-structured interview guides and thematic analytical frameworks were used to collect and analyze the data, respectively.

**Results:**

Availability of nutritious foods, strong social support from health care personnel, and lactating mothers’ awareness of healthy eating practices emerged as the enablers of healthy eating among lactating mothers. The barriers include limited income, seasonal availability of fresh foods, food myths and taboos, and patriarchal practices.

**Conclusions:**

These findings have implications for maternal and child nutrition practice and policy in developing settings. An integrated development approach is needed to expand and sustain awareness of the importance of optimal nutrition for maternal and child health, ensure year-round local food production, and implement interventions that aim to remove the barriers to women’s dietary decision-making autonomy in the study setting and beyond.

## Introduction

The health status of the lactating mother influences the child’s health, as the nutrients in breast milk and the mother’s overall physical and emotional wellbeing during lactation have implications for the infant’s early health and their health in later life [[1], [2], [3]]. Lactation refers to the secretion of milk from the mammary glands of a mother that occurs immediately after childbirth [[4], [5], [6]]. The Ghana Demographic and Health Survey (GDHS) 2022 data shows that the national exclusive breastfeeding rate for children aged <6 mo was ∼53%. In the Upper West Region where this study was carried out, ∼74% of children aged 0 to 1 mo were exclusively breastfed [7]. However, by age 2 to 3 mo, there is a decline to ∼65%, and by 4 to 5 mo, the percentage of children exclusively breastfeeding further reduces to 25% [7]. Optimal maternal nutrition is important, especially when mothers are practicing exclusive breastfeeding. Studies have shown that maternal dietary habits during breastfeeding may influence human milk composition. Mothers who consume foods rich in iron, calcium, vitamin A, and vitamin D tend to have better breast milk quality. On the contrary, nutrient deficiencies may impact breast milk composition and the health of mothers and infants [2,3,8]. This illustrates the importance of nutritious and well-balanced dietary patterns, such as the intake of fruits, vegetables, and whole seeds during lactation. Thus, optimal maternal nutrition not only promotes the mother’s health but also provides the infant with milk containing an adequate amount of nutrients for balanced nutrition and growth [2,3,8].

In recognition of the need to address the deficits in nutrition for lactating mothers and infants, Ghana has been implementing maternal and child nutrition policies and programs for some decades now. For example, the Baby-Friendly Hospital Initiative (BFHI) and the subsequent International Code of Marketing of Breast Milk Substitutes were initiated in 1995 and 2000, respectively. Following these, the National Nutrition Policy (2013–2017) (Ghana Health Service, 2013) and the Maternal and Child Health and Nutrition Improvement Project (2014–2020) have all contributed to improving the nutritional status of the population, especially lactating mothers and children (World Bank, 2014). This progress in improving nutrition for lactating mothers and children is reflected in the trend analysis of anthropometric measurements from previous GDHSs, which shows that there have been improvements in the nutritional status of children under age 5 in the last 30 y. For example, the prevalence of stunting decreased from 33% in 1993 to 17% in 2022, and the percentage of children who are wasted and underweight reduced from 14% in 1993 to 6% in 2022, and 23% in 1993 to 12% in 2022, respectively [7,9].

This notwithstanding, recent surveys have shown higher prevalence of anemia in women and children and stunting, wasting, underweight, and obesity in children in populations in underserved regions in Ghana [7]. The most recent GDHS found that 49% of children aged 6 to 59 mo and 41% of women aged 15 to 49 y are anemic, and anemia prevalence is 51% among pregnant women [7]. Oti Region and the 5 regions in the north of the country (Northern, Upper East, Upper West, Northeast, and Savana) recorded higher prevalence rates of anemia in women and children than the national average [7]. This skewed variation in nutrition statistics is consistent with the regional variation in the incidences of poverty in the country [10,11]. In the Upper West Region, where this study was conducted, the Wa West District is ranked highest in the incidence of poverty [10,12] and undernutrition among women and children [13].

This study draws on the socio-ecological theory to explore the enablers and barriers to healthy eating for lactating mothers in the Wa West District in Ghana. Previous studies on this subject found that the knowledge and practice of exclusive breastfeeding were influenced by health professionals, support and encouragement from family and community members, and environmental characteristics [14,15]. Other studies focused on the barriers to optimal nutrition for lactating mothers and identified negative cultural beliefs and practices, misinformation [[16], [17], [18], [19], [20]], poverty and food insecurity [17,18,20], and patriarchal norms [18,21,22] as the main barriers. Dalaba et al. [16] found that different cultural beliefs and taboos about what foods are considered healthy and unhealthy for women at different stages of the reproductive period were a barrier to healthy eating among lactating mothers in the Kassena-Nankana East Municipal in Ghana. Similarly, a review of literature on the barriers to maternal nutrition in low- and middle-income countries identified that cultural beliefs around the understanding of foods deemed unhealthy during lactation led to dietary restrictions. The study also found that food aversions, economic constraints, and household food availability influenced the dietary decisions of lactating mothers [20]. In a study on maternal and child nutrition in rural northern Ghana, Debpuur et al. [17] reported poverty, the lack of an irrigation system to facilitate year-round farming, and poor harvests as the main barriers to optimal nutrition. The study advocated support for community-initiated nutrition interventions such as improved irrigation for dry season farming, provision of agricultural inputs, and community education to improve maternal and child nutrition. Other studies observed that women’s adherence to nutrition advice was hampered by constrained autonomy resulting from patriarchal norms including spousal control, coercion, and financial abuse [18,21,22]. Despite this extensive research on the subject, the enablers and the opportunities for promoting optimal nutrition among lactating mothers and children in underserved rural settings have seldom been explored, although this focus is needed to inform the (re)design of nutrition policy and nutrition-enhancing interventions in the country and beyond.

This study broadens the scope of the discussion of healthy eating among lactating mothers and draws on the socio-ecological theory to explore the barriers and the opportunities for promoting healthy eating among lactating mothers in underserved settings. This holistic approach not only provides a better understanding of the issue but sets the stage for advocacy for a review of the National Nutrition Policy and guides the implementation of interventions to improve maternal and child nutrition in the country, particularly in deprived settings where the one-size-fits-all approach to nutrition policy design and implementation has not adequately addressed their peculiar nutrition needs.

## Theoretical Perspective: the Socio-Ecological Theory

The socio-ecological theory provides an appropriate framework for analyzing the barriers and enablers of healthy eating practices among lactating mothers. This theory asserts that factors at the individual, household, and community levels influence health behaviors and that there is a continuous and reciprocal interaction between individuals and their community of residence [14,23,24]. Thus, we draw on the socio-ecological theory to explore the barriers and enablers of healthy eating practices among lactating mothers. We argue that the barriers and enablers of healthy eating practices of lactating mothers are influenced by 3 key factors: individual-level attributes, household-level factors, and the larger community characteristics.

Individual-level attributes of the theory would include such variables as a lactating mother’s age, education, income status, and place of residence. According to Raut et al. [25], education influences users’ ability to access information on nutrition and healthy eating for lactating mothers and children. Additionally, women’s income status influences their ability and willingness to purchase the recommended foods and food supplements for consumption. Recent studies have shown that lactating mothers are not choosing healthy foods because of income poverty [26]. In terms of household-level factors, variables such as household members’ income and their educational attainment determine their ability to afford the cost of healthy foods.

Community characteristics may influence the eating habits of lactating mothers in a variety of ways. First, consistent with the tenets of this theory, scholars have argued that jointly increasing geographic and economic access to markets or farm stands increases vegetable intake among low-income groups [[27], [28], [29]]. Second, the population’s perception about foods that are deemed healthy and unhealthy for women at different stages of the reproductive period could significantly influence the eating habits and norms of lactating mothers. For example, a recent study in rural northern Ghana found that cultural beliefs held by the participants that the consumption of fatty foods and fresh meat increases the size of both the pregnant woman and the baby and could lead to complications during delivery [16]. Similarly, disparities in access to health information between urban and rural populations may alter the level of knowledge about healthy eating and dispel misconceptions regarding the consumption of certain foods. Additionally, structural gender inequities that characterize predominantly patriarchal communities, including women’s limited decision-making autonomy on the types of foods that are eaten by the household in general, may influence the attitudes and practice of healthy eating by lactating mothers [18,21,22]. These factors may play out differently in different communities because of variations in socio-cultural norms and value systems and thus shape lactating mothers’ adoption of healthy eating.

## Methods

### Study design

This article follows an earlier publication from a broader study on the barriers and enablers of healthy eating among lactating mothers in a poor rural district in northern Ghana [26]. Thus, similar to the initial publication, we employed an exploratory case study design to understand the hidden barriers as well as the enablers of healthy eating practices among lactating mothers in a resource-poor setting in north-western Ghana. A qualitative exploratory approach was appropriate as it enabled us to construct knowledge through a comprehensive understanding of participants’ lived experiences [30], particularly the dietary practices of lactating mothers.

### Study setting

Under the Local Government Act 463, 1993, the Wa West District was separated from the Wa District in 2004 by a legislative instrument (LI 1751). There were 51,077 females and 45,880 men among its 96,957 total residents. According to the 2020 Population and Housing Census report, every community in the district is rural and primarily impoverished [10]. With Wechiau as its capital, the district has borders with Sawla Tuna Karlba District to the south, Wa Municipal to the east, Nadowli Kaleo District to the north, and Burkina Faso to the west [31]. The Wa West District is relatively under-resourced [32] and has 1 hospital, 8 subdistrict health centers, and 40 functional Community Health Planning Services (CHPS) zones in contrast to the Wa Municipal, which has a regional hospital, a municipal hospital, 10 subdistrict health centers, and 47 functional CHPS zones [13,26]. Compared with other districts in the region, the population’s health status is poor, as evidenced by 2023 data on childhood and maternal mortality, where infant mortality is 1.6 deaths per 1000 live births, under-five mortality is 5.8 deaths per 1000 live births, and neonatal mortality is estimated at 1.6 deaths per 1000 live births. The maternal mortality rate is 38.9 per 100,000, and 0.5% of children age <5 y are underweight [33]. While the largest segment of the region’s population (70.7%) is poor or extremely poor, there is a wide variation in the headcount rate of poverty among the districts and municipalities, with the Wa West District having the highest poverty rate (90%) [12]. Given this context of limited resources for health, pervasive poverty, and women’s limited decision-making autonomy, the district provides an appropriate setting to draw on the socio-ecological theory to explore for a deeper understanding of the barriers and the enablers of healthy eating among lactating mothers in the district.

### Sampling strategy

The district, health centers, lactating women, and health care professionals were selected using a multistage purposive sampling technique. Purposive sampling was used to select the participants who were most likely to provide appropriate and useful information to achieve the objective of the study, which is to understand the enablers and barriers to healthy eating for lactating mothers in underserved settings. The initial stage entailed sampling of the study district, followed by sampling of health institutions, health care professionals, and lactating mothers. In deciding on the district for the study, we analyzed key development indicators and discovered that the Wa West District is wholly rural and has the highest incidence of poverty in the Upper West Region [10,32]. The sociodemographic characteristics of the population have a substantial impact on their health behavior [34], particularly women’s dietary decision-making autonomy for healthy eating [26]. In the second step, we deliberately selected 6 maternity health centers from a total of 9 in the district. These maternity health centers were selected because they were designated “baby-friendly” facilities. Baby-friendly maternity facilities are health facilities that have documented their full adherence to the Ten Steps of the WHO and UNICEF-launched BFHI, as well as their compliance with the International Code of Marketing of Breast milk Substitutes [35]. Following the selection of the health centers, we selected and interviewed 6 midwives and 2 nutrition officers. These participants were purposively selected because of their regular interaction with lactating mothers and their in-depth knowledge and understanding of their nutrition-related enablers and barriers. For example, the midwives provided relevant information on lactating mothers’ awareness of the benefits of healthy eating, mothers’ limited dietary decision-making autonomy, and limited extended family support to women during lactation. The nutrition officers provided in-depth information on some of the barriers to healthy eating, which include the health status of the mother and the need to counsel them on the right alternative foods to eat, the seasonality of fresh nutritious foods, and the food substitutes available to mothers. The study enrolled 30 lactating mothers who volunteered and accepted to participate. We conveniently sampled them when they visited the health centers with their children for welfare clinics, counseling, vaccination, and other health needs. They provided information on their awareness of benefits of healthy eating and the individual, household, and community-level factors associated with healthy eating among lactating mothers. The information from these participants were crucial for understanding and analyzing the barriers and the opportunities for lactating mothers to practice healthy eating in the study setting.

### Data collection

The data for this study were gathered between April and May of 2024. The data were generated through semi-structured key interviews and review of the existing literature on the enablers and barriers to healthy eating among lactating mothers. Prior to data collection, 2 postgraduate students (1 female and 1 male), who are experienced in qualitative data collection, ethics in research, and data management, were retrained for 2 d on data collection protocols, techniques of qualitative interviewing, and ethics in research. Two authors, MKD and RA, are experienced public health researchers who provided technical reinforcement during the field work. MNM is a native of the study area and therefore facilitated access to the key informants. Semi-structured interviews with key informants were the primary source of information for this study. This approach made it possible to collect in-depth information for understanding and analyzing the barriers and opportunities for promoting healthy eating among lactating mothers in the district. In furtherance of the process, we developed semi-structured interview guides and used the same to generate information from the participants on the thematic areas of the study. Knowledge of the literature on optimal nutrition for mothers and children and RA’s extensive experience implementing nutrition-related interventions informed the design of the semistructured interview guides. To ensure the robustness of the instruments, the first drafts were pretested with 3 lactating mothers and 3 health care providers at non-study settings. Based on the feedback from the pretesting, we fine-tuned the instruments for the main data collection process with community nutrition officers, midwives, and lactating mothers. Interviews were conducted in English for those who were literate and were willing to respond in English and in Dagaare (the local language) for lactating women who did not speak English. The interviewing of participants was a joint undertaking among all authors. MKD was assigned to interview the 2 nutrition officers and 15 recruiting lactating mothers recruited for the study. MNM conducted interviews with 15 lactating mothers, and RA, due to the language barrier, was responsibile for interviewing the 6 midwives in English. Since MKD and MNM are both proficient in the local language spoken by the mothers, they were tasked with interviewing all the lactating mothers in the local language to ensure clarity and comfort during the interviews. The interviews lasted between 45 min and 1 h. While the lactating mothers and the midwives were interviewed at their respective health centers face-to-face, the nutrition officers were interviewed in their offices. All the interviews were audio-recorded with the permission of the participants.

### Analysis of the data

After the interviews, the audio-recordings were saved for transcription. The first author simultaneously translated and transcribed the interviews into English. The Dagaare translations were double-checked by 2 members of the research team to verify that no meaning was lost in the transcription process. A thematic framework [36] was deployed in the analysis of individual-level attributes, household-level factors, and the larger community characteristics that impede or promote the practice of healthy eating among lactating mothers in the study setting. The analysis involved the 6-step analytical process of Maguire and Delahunt [37] to familiarize ourselves with the data, generate initial codes, search for patterns and relationships between codes, and develop themes that reflect these patterns. To familiarize ourselves with the data, we became immersed in it through repeated readings of the interview transcripts and listened to the audio-recordings repeatedly for clarity and certainty. In the next stage, we identified meaningful segments of the data and labeled them with concise codes to represent our initial interpretations and perspectives. From this point, we searched for patterns and relationships between the initial codes. We did this by clustering the codes into broader categories based on shared meanings and connections. We then reviewed the initial themes and fine-tuned them to ensure they accurately reflected the data and were distinct from each other. At this stage, we merged similar themes and split themes we found to be overly broad. Next, we carefully defined and named each theme to represent the underlying meaning and its relationship to the research question. The final step involved presenting the themes with illustrative quotes from the data and discussing the broader implications of the findings.

### Ethical standards disclosure

This study was conducted according to the guidelines outlined in the Declaration of Helsinki, and all procedures involving research study participants were approved on 27 March, 2024 by the institutional review board of the Christian Health Association of Ghana (CHAG-IRB01022023). Written informed consent was obtained from all participants, and the first participant was interviewed on 2 April, 2024.

## Results

### Characteristics of the lactating mothers

The study included 30 lactating mothers, 2 nutrition officers, and 6 midwives. The characteristics of the lactating mothers are provided in Table 1. Their ages ranged between the 18 and 44 y. At the time of the study, 1 mother was single, and the rest were married.

**TABLE 1:** Characteristics of the lactating mothers

Variable	Frequency
Marital status	
Single mother	1
Married	29
Occupation	
Farmer	12
Trader	3
Formal employment	4
Weaving	3
Seamstress	2
Student	1
Unemployment	5
Level of education	
Tertiary (diploma/degree/postgraduate)	4
Secondary education	2
Basic level	15
No formal education	8
Other (specify): Muslim education	1
Religion	
Christian	23
Islamic	7
Ethnicity	
Waala	8
Dagaaba	16
Fulani	1
Brifour	3
Ashanti	2
Community	
Lassia	5
Wechiau	5
Poyentanga	5
Egu	5
Vieri	5
Dorimon	5

The largest proportion (*n* = 11) of the 30 mothers were between the ages of 15 and 24, with the smallest group being those who did not know their ages (*n* = 3). Twenty-nine (*n* = 29) mothers were married, and 1 (*n* = 1) being a single mother. Farming was the participants' primary economic activity and source of employment. Twelve (*n* = 12) of the mothers were farmers, and 1 (*n* = 1) was a student. The majority of them (*n* = 15) had basic school education. Twenty-three (*n* = 23) of the mothers were Christian, and the remaining 7 (*n* = 7) were Muslims. Several of the mothers (*n* = 16) were Daagabas. Only 1 lactating mother in the study was of Fulani ethnicity.

Two main themes emerged from the analysis: *1*) socio-cultural factors associated with healthy eating; and *2*) socio-economic barriers to healthy eating. These, and the associated subthemes, are summarized in Table 2 and described in the subsequent paragraphs. Some quotes are presented to support the findings in the subthemes.

**TABLE 2:** Broad themes and subthemes

Broad themes	Socio-cultural	Socio-economic barriers
Individual-level factors associated with healthy eating	Awareness of the benefits of healthy eating Existing health condition as barrier to healthy eating	Lactating mothers’ limited income is associated with low consumption of healthy foods
Household-level factors associated with healthy eating	Family support	Limited household income influences lactating mothers’ low consumption of healthy foods
Community-level factors associated with healthy eating	Seasonal availability of healthy food stuffs in the community Women’s limited dietary decision-making autonomy Myths and taboos of the community	—

### Individual-level factors associated with healthy eating among lactating mothers

Three subthemes emerged from the interviews in relation to the individual-level factors that influence lactating mothers’ healthy eating habits, namely lactating mothers’ awareness of the benefits of healthy eating, existing health conditions preventing them from eating certain healthy foods, and limited income.

#### Awareness of the benefits of healthy eating

As a first step, we explored lactating mothers’ knowledge and understanding of the intricacies of healthy eating. All 30 lactating mothers with whom we interacted were aware of the benefits of a balanced diet for themselves and their infants. They demonstrated awareness that a balanced diet provides nutrients, improves breast milk production, and enhances the healthy growth of babies. One of the mothers said this when quizzed about her knowledge of the right balance of food to eat when lactating:

“I am aware that when I am lactating, I must eat a mixture of protein and vegetables and take in a lot of fluids to stay healthy and for adequate breast milk production to feed my baby.” (Lactating mother, Vieri)

They stressed the correlation between optimal nutrition and quality breast milk production to ensure that babies feed adequately, are protected from infections and illnesses, and grow healthily. These participants were aware that the food they eat is passed on to the baby through the breast milk. A lactating mother from Lassia Tuolo had this to say about how healthy eating leads to healthy growth of the baby:

“When we (lactating mothers) eat a balance diet, we produce enough quantity and quality of breast milk for the baby to feed and grow well. We are told during postnatal care that good quality breast milk makes babies more intelligent.” (Lactating mother, Lassia)

However, a lactating mother who had anemia and was visiting the health center for the first time conceded that her participation in the nutrition counseling session had exposed her to the right foods to eat. This is what she said about the usefulness of the nutritional counseling sessions:

“I visited the health center, and I was told that my blood has reduced due to poor eating habits. So, I interacted with the nutritionist, and she recommended that I drink milo at least once every day in the morning for a month. Now, I am confident that my blood level will improve.” (Lactating mother, Egu)

The midwives confirmed these accounts when they agreed that an important service they provided to mothers during antenatal and postnatal care was teaching them how to prepare healthy and balanced meals. A midwife had this to say about lactating mothers’ awareness of the benefits of healthy eating:

“They (lactating mothers) know the benefits of healthy eating because we (midwives) make a conscious effort to demonstrate to the lactating mothers how to mix flours such as maize flour with beans flour, beans and millet, groundnut and millet, and many more. These various flour combinations are so nutritious and healthy for lactating mothers.” (Midwife, Wechiau)

#### Health status as a barrier to healthy eating

The health condition of the women rarely serves as a barrier to healthy eating among lactating mothers in the district. However, there are occasions when lactating mothers are unable to eat certain prescribed healthy meals because of ill health, especially when they have been operated upon during childbirth. Thus, the birth process can influence what they can or cannot eat. In some instances, they can be restricted to light foods until such a time they can switch to a normal diet. A nutrition officer had this to say:

“When a mother goes through cesarean section, it affects how she eats. This effect just a small number of them in the community. There are also some lactating mothers, who as a result accidents are affected psychologically, which influences their dietary lifestyle.”

### Household-level factors associated with healthy eating

The subthemes that emerged with respect to the household-level factors associated with healthy eating by lactating mothers are limited household income and family support.

#### Limited household income

The incomes of households in the district are extremely low, and only 4 of the 30 lactating mothers who participated in this study worked in the formal sector and earned salaries of approximately Ghanaian cedi (GH¢) 2,000.00 per month. Most of the participants were seamstresses, weavers, farmers, and petty traders, earning between GH¢30 and GH¢50 per month. Lactating mothers who are unemployed rely solely on irregular remittances from their husbands to buy food and soup ingredients. Mothers who earned between GH¢30 and GH¢50 per month conceded that their income was not enough to adequately feed them during lactation. As a result, they would usually rely on their husbands and some family members for financial support. A lactating mother in the Dorimon community had this say about her inability to afford healthy meals due to limited household income:

“We are farmers (peasant) and our farming is seasonal. So, the only time we have disposable income is in October and November when we have sold cereals harvested from our farm. Beyond this period, we struggle to get money to buy healthy food stuffs.”

This statement was confirmed by the midwives. One of them indicated that:

“There is so much poverty in this area, and this situation makes it difficult for them to adhere to the right menu of healthy meals for lactating mothers.”

#### Immediate and extended family support

Given the pervasiveness of poverty in the district, receiving support from family members is one reliable source of healthy eating for lactating mothers. The support they received was either in the form of money or as food staples. The following are some quotes from the participants on family support toward healthy eating by lactating mothers:

“I receive support from both immediate and extended family. My husband gives me money to buy the food ingredients. My mother-in-law also sends me some of the food ingredients so that I can use to prepare the food.” (Lactating mother, Wechiau)

However, there are some women who do not receive any support from their families during the period of lactation. According to one of the mothers:

“I do not get any support from my family. After harvesting, I don’t get anything from them. They do not give me anything from the harvest to depend on.” (Lactating mother, Poyentanaga)

The nutrition officers and midwives discussed the close connection between childbirth and motherhood, and the traditions of support and care. They noted that while women receive support from their families, especially from their husbands, this assistance is temporal and not sustainable throughout the lactation period. One of the midwives, reflecting on the women’s experiences, had this to say:

“The support comes at the early stages of lactation, mostly during outdooring and the first few weeks. So, when that elapses that support also ends. They only support for the first week and after giving the mother meat for this short period, they stop giving for the rest of the lactation period.” (Midwife, Dorimon)

This was reiterated by a nutrition officer:

“The support from spouses is very low. Some say that it is the tradition. It is something that is running through history whereby it is the man’s responsibility to marry the woman, and she takes responsibility for feeding the household.” (Nutrition officer, Wechiau)

### Community-level factors associated with healthy eating

Three subthemes emerged with respect to the broader community-level factors associated with healthy eating among lactating mothers in the district, namely the limited availability of food stuffs in the community, women’s limited dietary decision making, and food myths and taboos of the community.

#### Seasonal availability of food

The main reason for the shortage of certain healthy foods in the Wa West District is seasonal availability. This is caused by the erratic and seasonal rainfall pattern experienced in the northern part of Ghana. This part of the country usually experiences 2 seasons: the dry season and the rainy season. The rainy season is often characterized by enormous food availability, making it possible for lactating mothers to secure the right balance of meals. However, the dry season presents the challenge of limited or very expensive fresh foodstuffs including fruits and vegetables. Their limited availability and high cost make them inaccessible to the poor, and their intake is very low. The participants concurred that the limited availability or unavailability of certain essential foods in the dry season reduces the consumption of healthy foods by lactating mothers. Below are some narrations from the interviews:

“Vegetables and fruits such as oranges and watermelons are very expensive during the dry season. I can hardly afford to pay for them even though the nutrition officer has counseled me to eat a lot of them for this lactation period.” (Lactating mother, Vieri)

Similarly, a nutrition officer shared this sentiment by indicating that:

“Foods over here are seasonal, and the people also eat what is available and affordable. For instance, there are plenty of fresh vegetables at affordable prices during the rainy season. However, during the dry season they become very expensive because the supply is very limited.” (Nutrition officer, Dorimon)

#### Women’s limited dietary decision-making autonomy

The saying, “We eat what we grow,” perhaps sums up the limited influence lactating mothers have in relation to dietary decision making in households in the study setting. Like all communities in northern Ghana, the communities in the Wa West District are characterized by patriarchal practices where men decide the types of crops cultivated on the farm or backyard garden. Additionally, men control the household funds, and decisions about the protein that accompanies the meal are at their discretion. Women’s lack of decision-making power in the household was cited as a key impediment to lactating mothers adhering to the healthy eating regimens prescribed by the nutrition officers. A lactating mother who described her husband as an embodiment of patriarchy said this:

“He is never interested in the carbohydrate component of the meal, rather, he is always interested in the type of protein that accompanies it, and so he dictates the type of meat or fish we eat, irrespective of whether I am lactating or not.” (Lactating mother, Egu Community)

This narration is corroborated by a statement by a midwife in the study:

“The men in this area do not allow their wives to make decisions about meals. They (men) decide on the cereals cultivated on the farms and would not provide funds for the purchase of vegetables and proteins (such as meat, eggs, and fish).” (Midwife, Wechiau Health Centre)

### Food myths and taboos

The responses on food myths and taboos produced mixed results. We were interested in finding out whether there are any foods that lactating mothers in the community are not allowed to eat. None of the 30 (*n* = 30) lactating mothers could name a food that they are prohibited from eating. The following interview extract supports this assertion:

“At the health facility we are encouraged to take the TZ, vegetable soup, rice and the soup and other meals like rice with stew etc. Our husbands have not prevented us from eating any meals that have been prescribed us by the nutrition officers” (Lactating mother, Vieri)

However, the 2 nutrition officers that participated in the study disagreed with this assertion and insisted that there were some food taboos that impede healthy eating in the community.

“People in this community do not eat snails because it is a taboo. For some of them, it is a taboo to take in nutritious proteins like fresh meat. They do this for spiritual purposes known to them alone. There are a lot of misconceptions attached, and we have been trying to demystify these myths and convince them to eat healthy foods.”

## Discussion

The period of lactation is characterized by high nutritional demands by both mothers and children. Breast milk must provide adequate nutrition for the infant during this period, and mothers are therefore required to eat healthily to meet the energy and micro and macronutrient requirements for sufficient breast milk production. The inability of mothers to meet their nutritional needs poses a threat to their health, as well as the health and wellbeing of the child. Adequate nutrition is also essential for sustained exclusive breastfeeding [2,3,38]. In this study, we explored the enablers and inhibitors of healthy eating among lactating mothers in a poor rural setting in Ghana. Exploring these enablers and inhibitors is important, as it provides context-specific evidence that can inform policies and programs that are aimed at improving the nutritional needs of women in resource-poor areas.

In terms of enablers, we found seasonal access to affordable, nutritious food, strong social support from health care providers, and adequate knowledge about the importance of a balanced diet during lactation as important enablers. The inhibitors to the achievement of optimal nutrition include low income, seasonal availability of nutritious foods, myths and food taboos in the community, and patriarchal practices that limit women’s autonomy in nutrition-related decision making. This section presents a discussion of the results of the study and the implications for maternal and child nutrition practice and policy.

The availability of nutritious foods such as fresh vegetables and fruits in the market during the rainy season is perhaps the most fundamental enabler of healthy eating among lactating mothers in the district. Participants reported that the availability of a wide variety of nutritious foods in the market at affordable prices makes it possible for pregnant women to adhere to the prescribed regimens for balanced diets. This finding is consistent with the perspective of the socio-ecological theory [24] and related scholarly works [27,28] that propose that community characteristics, such as easy access to resources such as markets to buy healthy foods, promote the consumption of nutritious foods and healthy eating practices in general. The necessity for year-round availability of fresh fruits and vegetables to ensure continuous intake of healthy foods calls for the establishment of irrigation systems to increase and sustain availability in the district and other locations where the rainfall pattern is erratic and seasonal. Eating fruits and vegetables during this period is optimal for breastfeeding as it enhances breast milk composition [2,3,8]. They provide a source of micronutrients such as vitamin A, vitamin C, and folate, which are essential for strengthening the immune system, and can protect the nutritional status of lactating women [2,3,8].

The other enabler manifests in the majority of the participants demonstrating awareness of the intricacies of healthy eating, including their knowledge of a balanced diet and their familiarity with the types of foods needed to enhance breast milk composition. They demonstrated awareness that quality breast milk protects the infant from infection and inflammation. This awareness was gained from the counseling interactions they had with the midwives and nutrition officers during welfare clinic visits. On the contrary, a lactating mother who had been diagnosed with anemia and was visiting the health center for the first time admitted to lacking knowledge of the recommended diet for pregnant women or lactating mothers. This illustrates the point that the creation of awareness of optimal nutrition for lactating mothers by the midwives and community nutrition officers not only serves as an important enabler of healthy eating among lactating mothers, but it also crystalizes the argument of the socio-ecological theory that individual attributes, such as being educated, can promote awareness and adoption of healthy eating habits [[23], [24], [25],^39^].

In contrast to these enablers, there were barriers associated with healthy eating among lactating mothers, ranging from individual and household attributes to community-level factors. First, a contributing factor to mothers’ awareness of healthy eating is family support during the period of lactation [14,15,40]. Ford et al. [40] argue that these socio-economic confounders influence the practice of healthy eating of lactating mothers. However, social support for lactating mothers from immediate and extended family members was often limited and temporal and therefore was identified as a barrier to healthy eating among mothers in the study setting. Previous studies found a positive correlation between social support and longer planned breastfeeding duration [14,15,[41], [42], [43]]. In the study setting where the extended family system is in practice and poverty remains widespread [10,26,32], strong social support is analyzed as an important instrument for promoting healthy eating among lactating mothers. The District Health Secretariat should plan and establish social support networks, incorporating spouses, lactating mothers, and mothers-in-law into breastfeeding training programs to help lactating mothers leverage these resources effectively.

Another important barrier facing lactating mothers in their quest to incorporate the habit of healthy eating is limited income. The results show that lactating mothers, although aware of the right mix of meals to eat, are sometimes unable to secure them because of low household income. Consistent with evidence of extreme poverty in the study district [10,12,26,32], we found that households’ low purchasing power constrains their ability to afford the recommended diets, particularly in the dry season when essential food items, such as vegetables and fresh fruits, are either very expensive or are not available in the community. Poverty, as a barrier to optimal nutrition for lactating mothers in northern Ghana, was a key finding in previous studies [[16], [17], [18],^20^,^26^]. These studies recommended interventions that will empower households economically and increase their purchasing power.

The issue of food myths also emerged in the study as a constraining factor preventing the consumption of healthy diets by lactating mothers. This arises from the cultural belief that proteins like snails and fresh meat are taboo, and pregnant women are prohibited from eating them. We argue that as they are already trapped in the shackles of poverty and limited availability of fresh foods, the prevalence of food myths and taboos is unlikely to enhance the nutrition statuses of women and children. The finding is consistent with observations made in previous studies in developing settings that fatty foods and fresh meat are taboo for pregnant women because of the belief that they increase the weight of both the pregnant woman and the baby, which can lead to delivery complications [16,19,44]. The continuous prevalence of food myths and taboos in rural communities calls for the intensification and expansion of culturally friendly education and awareness of the benefits of optimal nutrition for maternal and child health.

Perhaps the most profound yet hidden barrier to healthy eating among lactating mothers in the study setting is the prevalence of patriarchal practices in patrilineal marriage systems. This manifests in a variety of ways, including men’s dominance in decision making about the types of crops that are cultivated on the farms and their control over the household’s resources. A lactating mother’s remark that “we eat what we grow” aptly sums up women’s limited dietary decision-making autonomy in this setting. Although lactating mothers are aware of the recommended diets, decisions about their meals are often influenced by the farm produce of the household. This patriarchal practice limits women’s decision-making autonomy on the types of foods that are eaten by the household, and this may influence the healthy eating habits of lactating mothers. Previous research revealed that women in patrilineal tribes in northern Ghana play a subordinate role in the ownership and control of household resources [18,21,22,45]. To address the negative impact of patriarchy on maternal and child nutrition, we advocate the implementation of interventions that address systemic issues relating to women’s agency, which are necessary for achieving greater gender parity in heteropatriarchal settings in the long run. This is important because gendered inequalities serve to deepen the deleterious consequences of poverty [46]. Thus, to fight poverty is to undo these gender-related inequalities. Indeed, it is in recognition of the influential role of this in the achievement of sustainable development that the Sustainable Development Goals (SDGs) focus on empowering women and achieving gender equality.

In conclusion, the findings of the study reflect evidence of both enablers and barriers associated with healthy eating among lactating mothers. Lactating mothers’ awareness of healthy eating practices, the availability of nutritious foods, and the availability of nutrition and midwifery services in the district have been identified as the key enablers of healthy eating among lactating mothers. These notwithstanding, the pervasiveness of negative sociodemographic characteristics, such as limited household income, limited family family, seasonal availability of fresh fruits and vegetables, food myths and taboos, and patriarchal practices, have proved to inhibit healthy eating among lactating mothers in the district. As these findings have implications for maternal and child health and the implementation of SDGs 1 (No poverty), 2 (Zero hunger), 3 (Good health and wellbeing), and 5 (Gender equality), we advocate the deployment of an integrated development approach that will involve expanding and sustaining awareness of the importance of optimal nutrition for maternal and child health in the district, improving local food production through irrigation development, and implementing interventions that aim to achieve greater gender parity in the long run.

### Limitations of the study

Our choice of a qualitative approach made it possible to explore in-depth the barriers and the enablers of healthy eating among lactating mothers in the district. In this regard, although we managed to generate in-depth information that answered the research question, it must be acknowledged that the data was generated in one district in the country. Thus, the findings represent the views of purposively selected participants from a limited number of health facilities in the Wa West District. For this reason, the findings of the study are merely indicative of the barriers and facilitators of healthy eating among lactating mothers in resource-poor settings and are not meant to be generalized to represent the situation in the entire country and beyond. Additionally, the study did not provide data on the age of the children and their feeding status to clarify the direct relevance of maternal nutrition to breastfeeding outcomes. We therefore acknowledge this as a limitation and an opportunity for future research.

## Author contributions

The authors’ responsibilities were as follows – MKD: formulated the research question(s), designed the study, analyzed the data and wrote the article; MNM: designed the study, carried out the data collection, analyzed the data, and wrote sections the article; RA: analyzed the data and wrote sections of the article; and all authors: read and approved the final manuscript.

## Funding

The authors reported no funding received for this study.

## Conflict of interest

The authors report no conflicts of interest.
